# Acute Flaccid Myelitis in COVID-19

**DOI:** 10.1259/bjrcr.20200098

**Published:** 2020-07-24

**Authors:** Mohamed Abdelhady, Ahmed Elsotouhy, Surjith Vattoth

**Affiliations:** 1 Neuroradiology Departement, Neuroscience Institute, Hamad Medical Corporation, Doha, Qatar; 2 Adjunct Assistant Professor of Clinical Radiology, Weill Cornell Medicine-Qatar, Doha, Qatar; 3 Associate Professor of Clinical Radiology, Weill Cornell Medicine-Qatar, Doha, Qatar

## Abstract

Spinal cord imaging findings in COVID-19 are evolving with the increasing frequency of neurological symptoms among COVID-19 patients. Several mechanisms are postulated to be the cause of central nervous system affection including direct virus neuroinvasive potential, post infectious secondary immunogenic hyperreaction, hypercoagulability, sepsis and possible vasculitis as well as systemic and metabolic complications associated with critical illness. Only a few case reports of spinal cord imaging findings are described in COVID-19, which include transverse myelitis, acute disseminated encephalomyelitis and post-infectious Guillain Barre’ syndrome. We are describing a case of myelitis which, to the best of our knowledge, is the first reported case of myelitis in COVID-19.

## Case presentation

**Figure 1.**
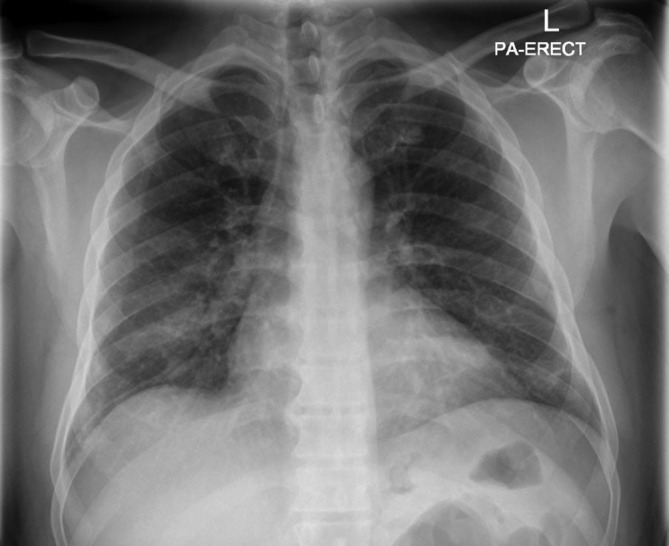
Frontal chest radiograph displays bilateral scattered lung infiltrates most pronounced in right lower lung zone.

**Figure 2.**
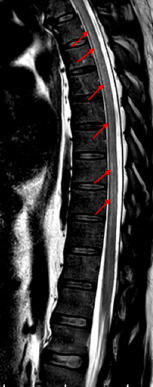
Sagittal *T*2 weighted image MRI shows slight hyperintensities in the ventral aspect of the upper and mid-thoracic cord (arrows).

**Figure 3.**
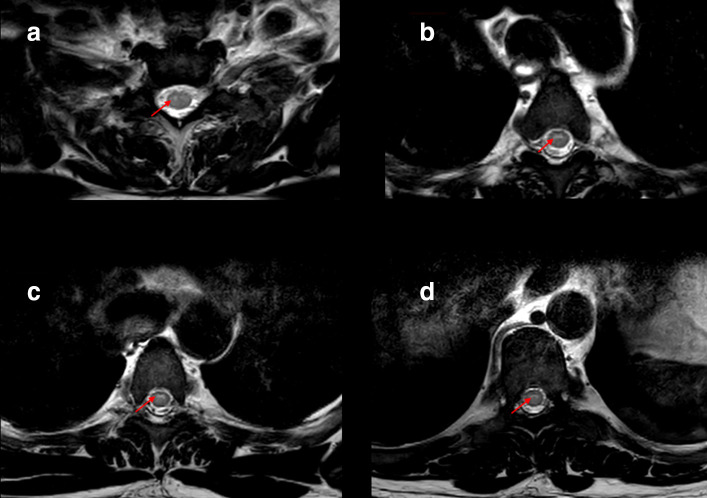
Axial *T*2 weighted images MRI at the level of upper thoracic cord (a, b) and at the level of mid thoracic cord (c, d) show hyperintensities in the ventral horns (arrows) of upper and mid thoracic cord bilaterally.

**Figure 4.**
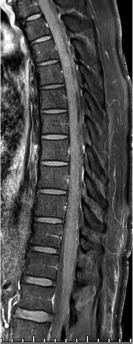
Sagittal post-contrast fat-saturated *T*1 weighted image MRI shows no evidence of abnormal enhancement in the affected segments of the cord.

A 52-year-old male presented with lower abdominal pain and inability to pass urine for the past 3 days, associated with fever and bilateral lower limb weakness (flaccid paralysis). There was no sensory affection. He was a known case of type II diabetes mellitus and G6PD deficiency. Routine admission chest radiograph of the patient showed bilateral scattered lung infiltrates most pronounced in the right lower lung zone (Figure 1). Patient was then tested positive for COVID-19 PCR in nasopharyngeal and oropharyngeal swabs. MRI of the brain and spinal cord was requested to further evaluate the lower limb weakness. Brain MRI was normal, but the spinal cord MRI displayed a continuous long segment of *T*2WI hyperintensity in the ventral horns of grey matter in the upper and mid-thoracic cord with no intervening normal cord (Figures 2 and 3). Remainder of the grey matter in the involved segments and the spinal cord white matter were normal. There was no evidence of post-contrast enhancement (Figure 4). The imaging features were suggestive of viral myelitis.

Lumbar puncture was performed which showed cerebrospinal fluid (CSF) increased white blood cells (WBCs) preferentially lymphocytes and increased proteins; further supporting the MR diagnosis of viral myelitis. Extensive laboratory work-up including CSF culture, blood culture, autoimmune screening (ANCA & ANA), Tuberculosis PCR and viral (hepatitis including B and C as well as herpes simplex viruses) serology were done, which all turned out negative. The complete blood count showed increased WBCs and lymphocytosis.COVID-19 PCR from CSF done on the third day of symptoms was negative, however the negative virus PCR in CSF does not exclude the diagnosis of viral myelitis as there is higher probability of false negative test results if the CSF PCR was done before the fifth day of symptoms.^1^ The constellation of the clinical findings, MRI features, laboratory data, and CSF findings are suggestive of viral myelitis most probable COVID-19 virus as it was the only detected virus after extensive work-up. Patient received steroids and acyclovir (antiviral drug). Unfortunately, two days following MRI, the patient developed cardiac arrest and eventually died. Hence, repeat CSF PCR during the period of peak positivity at fifth day of symptoms could not be done.

## Discussion

The ventral horn pattern of affection in our case favours viral myelitis over the broader spectrum term of transverse myelitis which encompasses infectious, para-infectious and non-infectious entities.^2^ Transverse myelitis has, in contradistinction to our case, centromedullary pattern of affection displaying intramedullary *T*2 weighted images hyperintensity of elliptical morphology which could affect the whole transverse extent of the spinal cord on axial images.^2^ Acute infectious transverse myelitis is usually caused by herpes simplex virus Type 2, varicella zoster virus (VZV), Epstein-Barr virus (EBV) or cytomegalovirus (CMV), while infectious long segment spinal cord affection is more typically observed with flaviviruses and enteroviruses.^1^

The ventral horn pattern of affection was previously described with many other non-pandemic viruses including poliomyelitis, flaviviruses (West Nile virus,dengue virus, Japanese B encephalitis and tick-borne encephalitis virus) and enterovirus 71.^1–3^ There are other patterns of spinal cord affection in infectious myelitis;the centromedullary pattern of affection is seen with EBV, Lyme disease and hepatitis myelitis. The preferential affection of lateral column is seen in human T-cell lymphotrophic virus-1 (HTLV-1). The affection of dorsal column is noted in VZV, neurosyphilis and progressive multifocal leukoencephalopathy (JC virus). Human immunodeficiency virus (HIV) can affect both lateral and dorsal columns.^2^

The continuous long segment of cord involvement, isolated grey matter affection and normal brain MRI makes other differentials like demyelinating disease less likely. Multiple sclerosis usually affects short segments and predominantly involves the white matter.^4^ Neuromyelitis optica spectrum disorders can have longitudinally extensive spinal cord involvement but are usually seen surrounding the central canal of the spinal cord where aquaporin four receptors are in abundance.^5^

Other imaging differential diagnosis for spinal cord ventral horn affection would be cord infarction, however this does not fit clinically owing to the subacute presentation of the patient and CSF findings which suggested viral myelitis.^6^

## Learning points

- We are reporting a case of acute flaccid myelitis in a COVID-19 patient with negative extensive labortatory work-up, including serology and CSF testing, for other infections raising possibility of myelitis caused by COVID-19 further supporting the direct neuroinvasive potential theory of the virus.
- Differential diagnoses for spinal cord ventral horn affection include cord infarction and viral myelitis.
- Viral myelitis ventral horn pattern of affection was previously described with many other viruses including poliomyelitis, flaviviruses and enterovirus.
